# Supplementary material for: Identification of photoperiod-induced specific miRNAs in the adrenal glands of Sunite sheep (Ovis aries)
Source: Front Vet Sci. 2022 Jul 22;9:888207. doi: 10.3389/fvets.2022.888207 (PMC9354845; doi:10.3389/fvets.2022.888207)
Supplement: Supplementary file 1 [file Data_Sheet_1.ZIP › Supplementary file/miRDeep2 file.zip!/miRDeep2 file/Know_Structure/LP42A3/oar-mir-148a.pdf]

miRBase precursor : oar-mir-148a
Total read count : 2154281
oar-miR-148a read count : 2151190
remaining reads : 3091

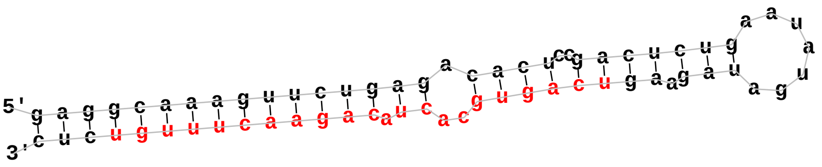

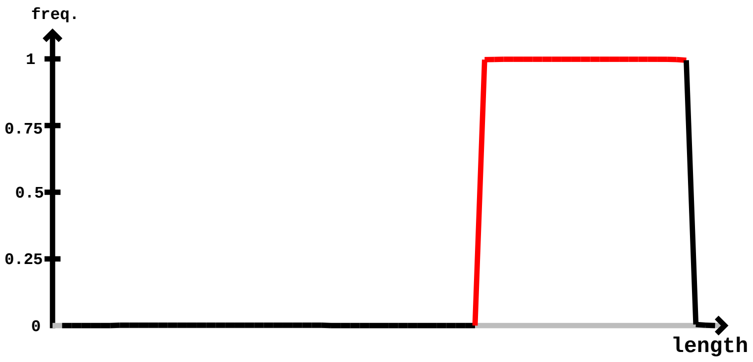

oar-miR-148a

5'- gaggcaaaguucugagacacuccgacucugaauaugauagaagucagugcacuacagaacuuugucuc -3' exp

((((((((((((((.((((..((((((.......))).))))))))..)).))))))))))))))	reads	mm	sample
....caaaguucugagacacuccga...	2	0	seq
....caaaguucugagacacuccgac..	1	0	seq
....caaaguucugagacacuccgacu...	64	0	seq
.....aaaguucugagacacuccg..	8	0	seq
.....aaaguucugagacacuccga...	84	0	seq
.....aaaguucugagacacuccCa...	1	1	seq
.....aaaguucugagacacuccgac..	24	0	seq
.....aaaguucugagacacuccgacu...	2888	0	seq
.....aaaguucugagacacuccgacuc..	7	0	seq
.....aaaguucugagacacuccgacucu.......................................	7	0	seq
.....aaaguucugagacacuccgacucug......................................	3	0	seq
......aaguucugagacacuccgacu...	1	0	seq
......................................agaagucagugcacuacagaacuuugu...	1	0	seq
....gucagugcacuacagaacuuu.....	1	0	seq
....gucagugcacuacagaacuuug....	1	0	seq
....guUagugcacuacagaacuuugu...	1	1	seq
....gucagugcacuacagaacuuugu...	82	0	seq
....gucagugcacuacagaacuuuguc..	2	0	seq
....ucagugcacuacagaac........	1	0	seq
....ucagugcacuacagaacu.......	113	0	seq
....ucaCugcacuacagaacu.......	2	1	seq
....uUagugcacuacagaacuu......	1	1	seq
....ucagugcacuacagaacuu......	305	0	seq
....ucaguAcacuacagaacuu......	2	1	seq
....ucagugcacuacagaacuuu.....	1812	0	seq
....uUagugcacuacagaacuuu.....	2	1	seq
....ucagugcacuacagaacuuug....	5669	0	seq
....uUagugcacuacagaacuuug....	16	1	seq
....ucagugcacuacagaacuuugu...	2128257	0	seq
....uUagugcacuacagaacuuugu...	3053	1	seq
....uUagugcacuacagaacuuuguc..	8	1	seq
....ucagugcacuacagaacuuuguc..	5760	0	seq
....ucagugcacuacagaacuuugucu.	3084	0	seq
....ucagugcacuacagaacuuugucuc	12	0	seq
....cagugcacuacagaacuuu.....	1	0	seq

oar-miR-148a

```
gaggcaaaguucugagacacuccgacucugaauaugauagaagucagugcacuacagaacuuugucuc
............................................cagugcacuacagaacuuug....    3      0     seq
............................................Uagugcacuacagaacuuugu...    10     1     seq
............................................cagugcacuacagaacuuugu...    1261   0     seq
............................................cagugcacuacagaacuuuguc..    4      0     seq
............................................cagugcacuacagaacuuugucu.    2      0     seq
.............................................agugcacuacagaacuuug....    6      0     seq
.............................................agugcacuacagaacuuugu...    1688   0     seq
.............................................agugcacuacagaacuuuguc..    9      0     seq
.............................................agugcacuacagaacuuugucu.    3      0     seq
..............................................gugcacuacagaacuuugu...    2      0     seq
...............................................ugcacuacagaacuuugu...    3      0     seq
................................................gcacuacagaacuuugu...    14     0     seq
```
